# The draft genome of the carcinogenic human liver fluke *Clonorchis sinensis*

**DOI:** 10.1186/gb-2011-12-10-r107

**Published:** 2011-10-24

**Authors:** Xiaoyun Wang, Wenjun Chen, Yan Huang, Jiufeng Sun, Jingtao Men, Hailiang Liu, Fang Luo, Lei Guo, Xiaoli Lv, Chuanhuan Deng, Chenhui Zhou, Yongxiu Fan, Xuerong Li, Lisi Huang, Yue Hu, Chi Liang, Xuchu Hu, Jin Xu, Xinbing Yu

**Affiliations:** 1 Department of Parasitology, Zhongshan School of Medicine, Sun Yat-sen University, 74 Zhongshan 2nd Road, Guangzhou, 510080, PR China; 2 Key Laboratory for Tropical Diseases Control, Sun Yat-sen University, Ministry of Education, 74 Zhongshan 2nd Road, Guangzhou, 510080, PR China; 3 Guangzhou iGenomics Co., Ltd, 135 West Xingang Road, Guangzhou, 510275, PR China

## Abstract

**Background:**

*Clonorchis sinensis *is a carcinogenic human liver fluke that is widespread in Asian countries. Increasing infection rates of this neglected tropical disease are leading to negative economic and public health consequences in affected regions. Experimental and epidemiological studies have shown a strong association between the incidence of cholangiocarcinoma and the infection rate of *C. sinensis*. To aid research into this organism, we have sequenced its genome.

**Results:**

We combined *de novo *sequencing with computational techniques to provide new information about the biology of this liver fluke. The assembled genome has a total size of 516 Mb with a scaffold N50 length of 42 kb. Approximately 16,000 reliable protein-coding gene models were predicted. Genes for the complete pathways for glycolysis, the Krebs cycle and fatty acid metabolism were found, but key genes involved in fatty acid biosynthesis are missing from the genome, reflecting the parasitic lifestyle of a liver fluke that receives lipids from the bile of its host. We also identified pathogenic molecules that may contribute to liver fluke-induced hepatobiliary diseases. Large proteins such as multifunctional secreted proteases and tegumental proteins were identified as potential targets for the development of drugs and vaccines.

**Conclusions:**

This study provides valuable genomic information about the human liver fluke *C. sinensis *and adds to our knowledge on the biology of the parasite. The draft genome will serve as a platform to develop new strategies for parasite control.

## Background

*Clonorchis sinensis*, the oriental liver fluke, is an important food-borne parasite that causes human clonorchiasis in most Asian countries, including China, Japan, Korea, and Vietnam [1-3]. Increasing epidemiological evidence demonstrates the great socio-economic impact of this neglected tropical parasite, which afflicts more than 35 million people in Southeast Asia and approximately 15 million in China alone [1,4]. The origin of most clonorchiasis cases is the consumption of raw freshwater fish containing *C. sinensis *metacercariae, which excyst in the duodenum and then migrate from the common bile ducts to the peripheral intrahepatic bile ducts of their host [5]. Although clinical manifestations are often asymptomatic, repeated and chronic infections of *C. sinensis *can result in serious hepatobiliary diseases, including cholangitis, obstructive jaundice, hepatomegaly, fibrosis of the periportal system, cholecystitis, and cholelithiasis [6]. Most importantly, both experimental and epidemiological evidence strongly implies that liver fluke infection is one of the most significant causative agents of bile duct cancer-cholangiocarcinoma (CCA)-which is a frequently fatal tumor [7-10].

The life cycle of *C. sinensis *is complex and similar to that of *Opisthorchis viverrini*, involving asexual reproduction in an aquatic snail (myracidium, sporocyst, redia, and cercaria stages) and sexual reproduction in piscivorous mammals (adult worm stage). Mammalian hosts include humans, dogs, and cats [1,6]. *C. sinensis *adult worms establish themselves as parasites in the intrahepatic bile ducts and extrahepatic ducts of the liver, and they can even invade the mammalian gall bladder [3]. Long-term parasitism by liver flukes results in chronic stimulation of the epithelial cells of the bile ducts due to fluke excretory-secretory (ES) products, a variety of molecules released from parasites into the host bile environment [11]. Proteomic studies have identified the components of *C. sinensis *ES products that are thought to act as stimuli for host bile duct epithelium [12,13]. *In vitro *biochemical studies have indicated that ES products from liver flukes have important roles in feeding behavior, detoxification of bile components, and immune evasion [11]. For instance, granulin-like growth factor secreted by the carcinogenic liver fluke *O. viverrini *was shown to induce host cell proliferation, and the proliferative activity could be blocked by antibodies against granulin. These data indicate that secreted proteins, along with many other molecules, are released by parasites to induce local cell growth [14]. Transcriptome data sets for *C. sinensis*, which include substantial representation of ES products, also enable a better understanding of the mechanism of infection of this carcinogenic parasite [3].

Epidemiological studies in regions affected by liver flukes have shown a strong association between the incidence of CCA and the infection rate of parasites [6]. Despite the considerable impact of liver fluke-associated hepatobiliary diseases on public health, there are currently no effective strategies to combat CCA. This study provides genomic information for the carcinogenic human liver fluke *C. sinensis *based on *de novo *sequencing, and the draft genome described will serve as a valuable platform to develop new interventions for the prevention and control of liver flukes.

## Results and discussion

### *De novo *sequencing and genome assembly

To avoid assembly difficulties because of high heterozygosity, we extracted genomic DNA from a single adult fluke and constructed two paired-end sequencing libraries with insert sizes of approximately 350 bp and 500 bp. Two lanes of Illumina paired-end sequencing were performed for each library (Table S1 in Additional file 1); in total, we generated 94.3 million pairs of reads with an average read length of 115 bp. We screened out contaminants in the raw data, including 0.25% of raw reads mapping to the human genome (*Homo sapiens*) and 0.06% from *Escherichia coli*. No reads were detected from the cat genome (*Felis catus*). We made use of the Celera Assembler, which has been updated to enable the use of Illumina short reads of at least 75 bp in length. The Celera Assembler has been used in many genome assemblies, including the first whole genome shotgun sequence of a multi-cellular organism [15] and the first diploid sequence of an individual human [16]. By discarding the low quality ends, we trimmed the raw reads to 103 bp. We assembled the trimmed reads into 60,796 contigs with an N50 length of 14,708 bp, and we generated 31,822 scaffolds with an N50 length of 30,116 bp. We also sequenced the transcriptome of an adult fluke by Illumina sequencing with approximately 32 million paired-end reads with a read length of 75 bp. We then used RNAPATH [17] to construct 26,466 super-scaffolds with an N50 length of 42,632 bp (Table 1). The total length of the assembled genome is 516 Mb, approximately 20% smaller than the genome size estimated by *k*-mer depth distribution of sequencing reads (644 Mb; Figure S1 in Additional file 1; described in the Materials and methods section). The assembled genome does not contain any fragments of the mitochondrial genome [18], which may be due to the algorithm of the assembly software as this cannot successfully assemble extraordinarily high coverage regions, such as mitochondrial genomes. Among the reads left unmapped to the assembled genome, 0.4% could be aligned to the previously published mitochondrial genome with approximately 5,000× coverage using Bowtie [19].

**Table 1:** Summary of the *C.sinensis *genome assembly

	Total length (Mb)	Number	N50^a ^(bp)	N90^a ^(bp)	Longest (bp)
Contig^b^	515.56	60,796	14,708	4,079	137,874
Scaffold^b^	516.46	31,822	30,195	7,299	238,094
Super-scaffold^b^	516.47	26,446	42,632	8,441	400,764

^a^The N50 and N90 sizes of contigs or scaffolds were calculated by ordering all sequences and then adding the lengths from the longest to the shortest until the summed length exceeded 50% and 90% of the total length of all sequences, respectively. ^b^Contigs and scaffolds were constructed by Celera, while contigs were continuous sequence fragment without gaps (Ns). Super-scaffolds were built with RNA-seq data by RNAPATH based on the scaffolds.

The average GC content of the *C. sinensis *genome is 43.85%. Using non-overlapping sliding windows along the genome, we found a random distribution of sequencing depth over areas with different GC content (within a range of 30 to 60%) covering more than 99.9% of the genome sequence (Figure S2a in Additional file 1). Regions with lower (< 0.2) or higher (> 0.6) GC content were not found. The GC content of *C. sinensis *is higher than that of four other genomes that we examined (Figure S2c in Additional file 1).

To evaluate the single-base accuracy of the assembled genome, we mapped all of the trimmed reads onto the super-scaffold using Bowtie [19] (no more than three mismatches). Approximately 79% of the reads were uniquely mapped (Table S2 in Additional file 1). For more than 98% of the assembled genome, there are more than ten reads mapped for each position, and the maximum sequencing depth is 30× (Figure S2d in Additional file 1), which can provide a very high single-base accuracy [20]. To further evaluate the assembly accuracy, 14 pairs of primers were designed to amplify specific genomic fragments. All PCR products were sequenced on an ABI3730, and the resulting sequence traces aligned to the genome with over 99.6% identity (Table S3 in Additional file 1). The assembled genome contains 88.2% of the 15,121 ESTs produced by the Sanger method that have consensus lengths of 100 bp or more [21] (Table S4 in Additional file 1).

We called variants with the program glfSingle, which was designed for genome data from a single individual. We found 2.3 million variants (Figure S3 in Additional file 1), with a transition/transversion ratio of 2.07. The heterozygosity was approximately 0.4% for the whole genome, about three times that of *Schistosoma japonicum *[22].

### Repeat annotation

Several families of repeat elements covering 0.35% of the genome were identified by comparing the genome sequence with the known repetitive sequences in RepBase database. We further *de novo *predicted *C. sinensis*-specific repeats with the RepeatModeler software [23,24], and found 691 different repeat families/elements, constituting 25.6% (132.2 Mb) of the genome (Table S5 in Additional file 1). According to our estimate of genome size, approximately 128 Mb (19.9%) has not been assembled; most of the unassembled sequence may consist of repetitive sequences. The proportion of repeats is comparable to *S. japonicum *(40.1% [22]) and *Schistosoma mansoni *(45% [25]). We identified both non-long terminal repeats (non-LTRs) and LTR transposons, comprising 10.34% and 1.03% of the genome, respectively. Few short interspersed repetitive elements (SINEs) were found.

### Gene model annotation

Gene prediction methods (cDNA-EST, homology based, and *ab initio *methods) were used to identify protein-coding genes, and a reference gene set was built by merging all of the results. In total, we predicted 31,526 gene models (Table S6 in Additional file 1). To improve the accuracy of prediction, we considered gene models satisfying at least one of the following requirements as reliable: 1) gene function was annotatable; 2) genes were homologous to *S. japonicum *and *S. mansoni *genes; 3) genes were supported by putative full-length ORFs of *C. sinensis *(Table S7 in Additional file 1). In total, 16,258 gene models were retained as a reliable gene set and used for further analysis. Detailed analysis of gene length, exon number per gene and gene density in *C. sinensis *showed similar patterns to *S. japonicum *and *S. mansoni *(Table 2). Approximately 83.9% of the genes have homologues in the National Center for Biotechnology Information (NCBI) non-redundant database, and 57.8% can be classified with Gene Ontology terms [26]. Overall, 92% of the putative genes can be annotated (Table S7 in Additional file 1).

**Table 2:** General pattern of protein-coding genes of *C.sinensis *with *S. mansoni *and *S. japonicum*

	Number of gene models	Average gene length (bp)	Average protein length (bp)	Average exon length (bp)	Average number of exons	Averge intron length (bp)	CDS proportion (%)	Intron proportion (%)
*C. sinensis*	16,258	11,548	441	223	5.9	2,077	4.14	32.2
*S. japonicum*	12,657	9,999	392	222	5.3	2,059	3.70	28.00
*S. mansoni*	11,747	13,395	446	222	6	2,407	4.10	37.20

CDS, coding sequence.

To assess the completeness of our gene models, we investigated the coverage of the CEGMA [27] set of 458 core eukaryotic genes. Most of these core genes (425; 92.8%) were found, of which 392 were aligned over more than 50% of their sequences, suggesting the completeness of the genome (Table S8 in Additional file 1).

To investigate the amount of variation in gene families between *C. sinensis *and other metazoans, we assigned genes into families by clustering them according to their sequence similarities (see Materials and methods). We observed a minor amount of variation in the total number of gene families when looking across *C. sinensis *(6,910), *S. japonicum *(8,898), *S. mansoni *(7,313) and well characterized species like *Caenorhabditis elegans *(10,180), *Drosophila melanogaster *(7,640) and *Homo sapiens *(8,841) (Table S9 in Additional file 1).

**Figure 1:**
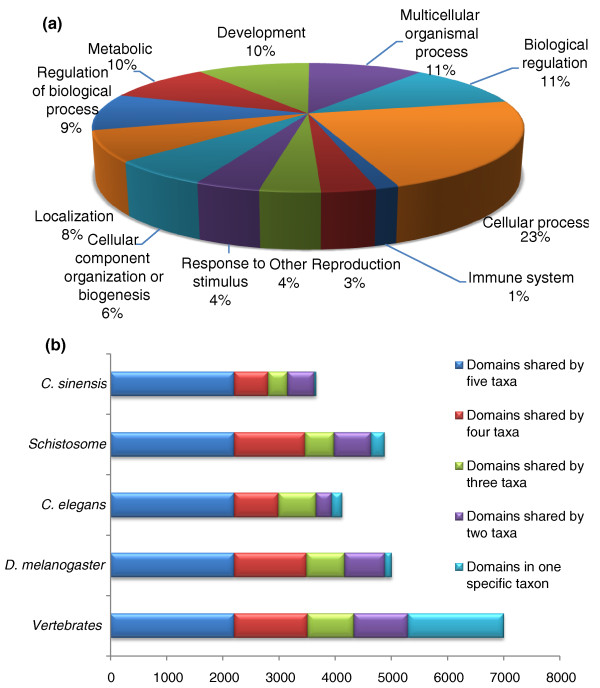
**Functional categorization of genes and protein domains of *C. sinensis***. **(a) **Proportions of the 9,371 *C. sinensis *proteins in different Gene Ontology categories (biological process terms only). The classification was carried out by CateGOrizer [28] based on the second level of the Gene Ontology category biological process. **(b) **8,371 domains were detected in *C. sinensis*, vertebrates (*H. sapiens*, *G. gallus *and *D. rerio*), *D. melanogaster*, *C. elegans *and *Schistosoma *(*S. japonicum *and *S. mansoni*). The major protein domains of *C. sinensis *are shared with other taxa and *C. sinensis *has the fewest unique domains.

Protein domains were identified by InterProScan (see Materials and methods). In total, 8,372 domains were found in the eight species (*C. sinensis*, *S. japonicum*, *S. mansoni*, *C. elegans*, *D. melanogaster*, *Danio rerio*, *Gallus gallus *and *H. sapiens*). Of the 16,258 gene models for *C. sinensis*, 6,847 contained a total of 3,675 protein domains (Table S10 in Additional file 1). Approximately 60% (2,203 of 3,675) of protein domains in *C. sinensis *were shared with other taxa (Figure 1), and these domains could be considered ubiquitous among metazoans. Among the 4,697 domains not detected in *C. sinensis*, 71% (3,345 of 4,697) were also not identified in *Schistosoma*. Only 29 domains present in *C. sinensis *and the other species mentioned were not in schistosomes. Thus, we speculated that domain loss events in *C. sinensis *might have occurred to an even greater extent than in *Schistosoma *(Figure S4 in Additional file 1). It is also possible that lack of completion of the draft genome could lead to an artifact of domain loss in *C. sinensis*. This conclusion needs further validation in our future work.

In addition to protein-coding genes, we also identified 7 rRNA fragments and 235 tRNAs, 509 small nucleolar RNAs, 169 small nuclear RNAs, and 858 microRNA (miRNA) precursor genes in the *C. sinensis *genome (Table S11 in Additional file 1). To further annotate *C. sinensis *miRNA precursors, we mapped miRNA expression data [28,29] to our miRNA precursors and found 159 miRNA precursors had evidence of expression (Additional file 2).

### Phylogeny of *C. sinensis*

**Figure 2:**
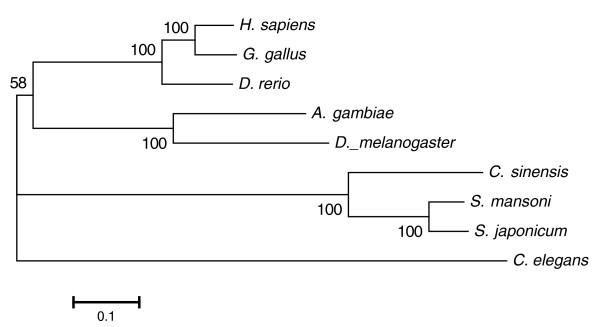
**Maximum likelihood phylogenetic tree**. The phylogenetic tree was constructed using concatenated amino acid sequences for 44 single-copy genes present in all nine genomes with maximum likelihood analysis. Numbers at the nodes indicate bootstrap values.

We used the *C. sinensis *sequences and eight other sequenced genomes to construct a whole genome-based species phylogeny. The eight additional species used to construct the phylogeny were *H. sapiens*, *G. gallus*, *D. rerio*, *D. melanogaster*, *Anopheles gambiae*, *C. elegans*, *S. mansoni*, and *S. japonicum*. The resulting phylogeny, based on 44 genes with single copy orthologues in all species, placed *C. sinensis *together with *S. mansoni *and *S. japonicum *(Figure 2). The topology structure of the tree is consistent with previous knowledge. *C. sinensis *and *S. mansoni *(or *S. japonicum*) were found to be evolving under a roughly constant evolutionary rate according to Tajima's relative rate test (*P *< 0.1).

### Synteny between *C. sinensis *and *S. japonicum *and *S. mansoni*

To investigate the long-range synteny between *C. sinensis *and the schistosome genomes, we selected all 79 scaffolds larger than 200 kb to perform alignments with the *S. japonicum *and *S. mansoni *genomes. Given that the average gene length of *C. sinensis *is about 10 kb, we chose those blocks with size larger than 10 kb as putative syntenic blocks. The largest syntenic block between *C. sinensis *and *S. japonicum *is 66 kb and the maximum gene number in one syntenic block is three. The largest syntenic block between *C. sinensis *and *S. mansoni *is 99 kb and the maximum gene number in one syntenic block is four (Additional file 3). More closely related species are needed to further understand the genome synteny of the flukes.

### Energy metabolism

To investigate energy metabolism in *C. sinensis*, we mapped the gene models to the pathways represented in the Kyoto Encyclopedia of Genes and Genomes (KEGG). The results demonstrate that both the glycolytic pathway (Figure S5 in additional file 4) and the Krebs cycle (Figure S6 in Additional file 4) are intact; *C. sinensis *can obtain energy from both aerobic and anaerobic metabolism. Although liver flukes inhabit anaerobic bile ducts, the conserved biochemical pathway of aerobic metabolism can facilitate the survival of *C. sinensis *juveniles in their intermediate hosts. As expected, genes encoding key enzymes required for glycolysis, such as hexokinase, enolase, pyruvate kinase and lactate dehydrogenase, were present at high copy number. We did notice that some genes for enzymes involved in energy metabolism were conspicuously absent; it seems that loss of these metabolic enzymes in *C. sinensis *might relate to its parasitic lifestyle. We presumed that *C. sinensis *adult worms might utilize exogenous glucose through the glycolytic pathway or by absorbing nutrients from hosts under anaerobic conditions [1]. Like schistosomes, *C. sinensis *can ingest glucose at rates as great as 26% of its body weight per hour, with glucose being broken down into lactic acid through glycolysis [30]. Thus, glycolytic enzymes are crucial molecules for trematode survival.

### Fatty acid metabolism and biosynthesis

**Figure 3:**
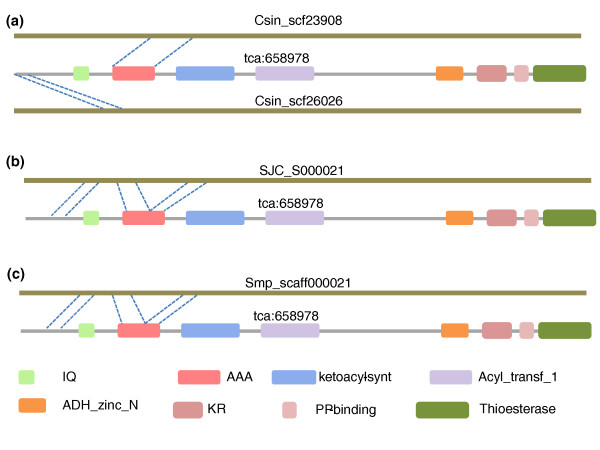
**Schematic diagram of the *FASN *gene (tca:658978) mapping to the *C. sinensis*, *S. japonicum *and *S. mansoni *genome**. All orthologues of fatty acid biosynthesis genes were BLAST to three fluke genomes. Only a fatty acid synthase (*FASN*) gene (tca:658978) could be significantly mapped (e-value < 1e-10), but two key domains of Ketoacyl-synt and Acyl_transf_1 were not observed in all of the three species. **(a) ***C. sinensis *(scaffolds: Csin_scf23908 and Csin_scf26026); **(b)***S. japonicum *(scaffold: SJC_000021). **(c) ***S. mansoni *(scaffold: Smp_scaff000021). AAA, ATPase family associated with various cellular activities; Acyl_transf_1, acyl transferase domain; Ketoacyl-synt, beta-ketoacyl synthase, N-terminal domain and C-terminal domain; KR, KR domain; Thioesterase, thioesterase domain.

After mapping gene models to KEGG pathways, we found fatty acid metabolism completely intact in *C. sinensis*, while fatty acid biosynthesis is lacking certain key enzymes. As indicated in Figure S7 of Additional file 4 all genes encoding enzymes necessary for fatty acid metabolism were found, but for the fatty acid biosynthesis pathway only three enzymes were detected: 3-oxoacyl-[acyl-carrier-protein] synthase II (FabF), acetyl-CoA carboxylase (EC 6.4.1.2, 6.3.4.14) and [acyl-carrier-protein] S-malonyltransferase (FabD) (Figure S8 in Additional file 4). To validate the gene losses in fatty acid biosynthesis, we searched for orthologous genes of this pathway in our gene models based on sequence similarity and domain organization. Only the three genes mentioned above resulted in reciprocal best BLAST hits and the same domain organizations (Additional file 5). To exclude the effect of incompleteness of the predicted gene set, we aligned all orthologous genes (excluding the three aforementioned genes) to the *C. sinensis *genome. Only one orthologue of *FASN *(encoding fatty acid synthase; KEGG gene ID tca:658978) got two significant hits. Detailed analysis of the two hits showed that two key domains (the beta-ketoacyl synthase N-terminal domain and the C-terminal domain) of *FASN *were not found (Figure 3a), suggesting these hits were not orthologues of *FASN*. Similar analysis was also performed in *S. japonicum *and *S. mansoni*, and the same results were observed (Figure 3b, c; Additional file 5). Since all three flukes have only the same three enzymes of the fatty acid biosynthesis pathway, it seems impossible that this pathway was lost by chance during sequencing and assembling of the three genomes by different techniques and laboratories [22,25]. Thus, we can conclude that the defect of fatty acid biosynthesis may have occured before the split of the three flukes.

We discovered many gene copies encoding fatty acid binding proteins, which are thought to have a role as fatty acid transporters in *Fasciola hepatica *[11]. Bile contains high levels of fatty acids, which can act as a nutrient source for parasites. The fatty acid binding proteins found in liver flukes may play an important role in the uptake of nutrients from host bile, possibly making it unnecessary for flukes to synthesize their own fatty acids endogenously. Niemann-Pick C1 protein (*NPC1*), a gene involved in regulating biliary cholesterol concentration, was also identified in *C. sinensis *[31]. The role of *NPC1 *in bile acid metabolic processes required for cholesterol absorption further indicates that *C. sinensis *is able to absorb lipids from its host for survival.

### Proteases, kinases, and phosphatases

To gain access to their preferred location within hosts, parasites have to escape hosts' defense mechanisms. Diverse molecules and biochemical pathways have evolved to counter those defenses, including important enzymes like proteases. Particularly in liver flukes, proteases play key roles in invasion, migration and feeding/nutrition [32,33]. Putative proteases we identified include metalloproteases, cysteine proteases, serine proteases and aspartic proteases, among others (Table S13 in Additional file 6). Among these, the largest group is the cysteine protease superfamily; these proteases have been identified as possible diagnostic antigens and vaccine candidates in *S. japonicum *[22]. Only those of the cathepsin F subtype are well characterized and theseare thought to play a key role in parasite physiology and related pathobiological processes in *C. sinensis *[34,35]. Other cysteine protease subtypes, such as cathepsins A, B, D, and E and even serine proteases, have not been previously recognized in *C. sinensis*, though they may contribute to catabolism of bilirubin and other host proteins. By comparing *C. sinensis *with *O. viverrini*, which has a similar life cycle, we were able to draw the general conclusion that serine proteases, metalloproteases and aspartic proteases may be principal players in host invasion and the progression of hepatobiliary disease [36-38].

Phosphorylation and dephosphorylation occur in all known eukaryotes through the antagonistic actions of protein phosphatases and protein kinases. Protein kinases play key roles in many eukaryotic processes, such as gene expression, metabolism, apoptosis, and cellular proliferation [39]. We have identified many important protein kinases in *C. sinensis *(Table S14 in Additional file 6), including casein kinase II, serine/threonine-protein kinase, cell division protein kinase, adenylate kinase isoenzyme, pyruvate kinase, cyclin-dependent protein kinase, calcium/calmodulin-dependent protein kinase, mitogen-activated protein kinase kinase kinase, and cAMP-dependent protein kinase. Casein kinase II is a eukaryotic serine/threonine protein kinase with multiple substrates and roles in diverse cellular processes, including differentiation, gene silencing, cell proliferation, tumor suppression and translation; however, its function in trematodes remains unknown [40]. Cyclic AMP-dependent protein kinase (PKA) is implicated in numerous processes in mammalian cells and plays an important role in parasite biology. Inhibition of *Plasmodium falciparum *PKA resulted in significant anti-parasitic effects [41]. Therefore, PKA represents a promising target for the treatment of parasite infections. Calcium/calmodulin-dependent protein kinase is essential for signal transduction in cells and modulates a variety of physiological processes, such as learning and memory, metabolism and transcription. For *Plasmodium gallinaceum *zygotes, calcium/calmodulin-dependent protein kinase is required for the morphological changes that occur during ookinete differentiation [42]. The mitogen-activated protein kinases (MAPKs) are highly conserved kinases involved in signal transduction and development [43]. In general, protein kinases are promising candidates as targets for RNA interference-based treatments to prevent liver fluke infection.

Apart from protein kinases, many phosphatases were discovered in the draft genome, including glucose-6-phosphatase 3, magnesium-dependent phosphatase and protein tyrosine phosphatase (Table S15 in Additional file 6). Phosphatases are endogenous kinase inhibitors that reverse the action of kinases, and they can be classified by substrate specificity as either serine/threonine, tyrosine or dual specificity phosphatases [44]. The physiological roles of serine/threonine protein phosphatases are numerous and have been studied extensively. Because of their critical regulatory roles in cellular processes, they have been regarded as promising targets for drug development in recent years.

### Tegument and excretory-secretory products

The outermost surface of a trematode is a syncytium. For platyhelminth parasites, the tegument is generally viewed as the most susceptible target for vaccines and drugs because it is a dynamic host-interactive layer with roles in nutrition, immune evasion and modulation, pathogenesis, excretion and signal transduction [45,46]. We characterized putative tegument proteins, including cathepsin B, epidermal growth factor receptor, glucose-6-phosphatase, glyceraldehyde-3-phosphate dehydrogenase, a calcium channel and a voltage-dependent channel subunit (Table S16 in Additional file 6). These proteins can be classified into several subtypes, such as proteases, receptors, nutrition and metabolism enzymes, channel proteins and transfer proteins. Most of these proteins have not previously been recognized in *C. sinensis *and may contribute to catabolism of host proteins and invasion of host tissue. Likely because of their critical roles, the genes encoding phospholipase D, phosphatidic acid phosphatase type 2A, glucose-6-phosphatase and calcium ATPase are found in high copy numbers. It is well known that phospholipase D is an important signaling molecule that increases nitric oxide synthesis and inducible nitric oxide synthase expression [47]. Phosphatase type 2A plays a pivotal role in the control of signal transduction by lipid mediators such as phosphatidate, lysophosphatidate, and ceramide-1-phosphate [48]. Our previous studies have revealed that some lipid metabolism enzymes, such as lysophospholipase [49] and phospholipase A2 [50], potentially contributed to liver fibrosis caused by *C. sinensis *infection. The roles of tegumental phospholipase D and phosphatase type 2A in *C. sinensis *pathogenesis warrant further study.

In the *C. sinensis *genome, we have identified some important ES products, including cortactin, aldolase, enolase, phosphoglycerate kinase, transketolase, programmed cell death 6 interacting protein, and fructose-bisphosphate aldolase (Table S17 in Additional file 6). The ES products of parasites have attracted attention because of their potential uses in the development of diagnostics, vaccines, and drug therapies. Previous studies have demonstrated the importance of ES products in many parasites, such as *O. viverrini*, *C. sinensis*, *S. japonicum*, *S. mansoni*, and *Paragonimus westermani *[45-48,51]. ES products comprise various proteins, the most predominant of which are proteases and detoxifying enzymes, which may serve vital roles in protecting parasites from host immune defenses [50]. One of the ES products, enolase, is a cytosolic glycolytic enzyme that has been reported to localize on the cell surface and the tegument in helminths. The secretory enolase of *S. japonicum *may promote fibrinolytic activity to enable parasitic invasion and migration within the host. This enzyme could be used for vaccines and drug development applications [47]. Similarly, fructose-bisphosphate aldolase is a conserved enzyme that was classified as a metabolic enzyme that modulates interactions between hosts and parasites [47]. This enzyme might have important roles in *C. sinensis*.

### Host-binding proteins and receptors

The highly co-evolved relationship between *C. sinensis *and its hosts depends on adaptations in the host-binding proteins and related receptors [52]. A number of such molecules were characterized in our research (Table S18 in Additional file 6), such as fibronectin, calmodulin, plasminogen, epidermal growth factor receptor and fibroblast growth factor receptor. Fibronectin, a multifunctional protein, is well conserved across species and has multiple domains for interaction with extracellular matrix components, such as heparin and collagen [53]. It was reported that fibronectin plays a role in activating phosphokinase A in the context of host invasion. Calmodulin has roles in the detoxification system, which has evolved sensors and responders that use Ca^2+ ^as a messenger. Plasminogen plays important roles in processes such as fibrinolysis and the degradation of extracellular matrices, and it can enhance proteolytic activity and increase tissue damage when coupled with its receptor. One of the most well characterized plasminogen receptors in mammals is enolase, the glycolytic enzyme described above [54]. Unexpectedly, a granulin-like growth factor was also observed (Table S18 in Additional file 6). This growth factor is a homologue of human granulin, a secreted growth factor associated with liver fluke-induced cancers [14]. Further studies should focus on the identification of host receptors to provide therapeutic strategies for cancers. *C. sinensis *can live for years, sometimes decades, within the bile ducts of mammalian hosts as it develops, matures and reproduces, so it is expected that co-evolution of parasite and host proteins has occurred in the process of regulating host-parasite interactions.

### Sex determination and reproduction

*C. sinensis *is a hermaphrodite, but the key genes responsible for sex determination are still unknown. We identified 53 genes related to sex determination, sex differentiation and sexual reproduction (Table S19 in Additional file 6). We also identified 25 genes in particular by their annotation with the Gene Ontology term 'hermaphrodite genitalia development'. That Gene Ontology annotation comes from *C. elegans*, a nematode that displays hermaphroditism [55]. In addition, six genes were predicted to be related to sexual reproduction.

In *C. sinensis*, we also found the genes *SOX6 *(*SRY *(sex determining region Y)-box 6) and *DMRT1 *(doublesex and mab-3 related transcription factor 1), which are known sex determination genes in vertebrates. In mammals, *SRY *is thought to be a testis determination factor and a critical developmental regulator [56]. The fact that *SRY *and *SOX6 *co-localize with splicing factors in the nucleus indicates that *SOX6 *may play a role in splicing of the testis-determining factor in *C. sinensis *development [56]. Doublesex and mab-3 contain a zinc finger-like DNA-binding motif (DM domain) that performs several related regulatory functions. *DMRT1 *regulates a DM-domain-containing protein that has a conserved role in vertebrate sexual development [57]. To date, most investigations of hermaphrodite development have focused on the nematodes, and our novel findings now provide valuable clues for biological research on the hermaphrodite phenomenon.

### Liver flukes and cholangiocarcinoma

Of particular interest in this study was the identification of proteins that could contribute to carcinogenesis. Apart from the previously described granulin and thioredoxin peroxidase, fatty acid binding protein and phospholipase A2 are members of the CCA-related gene group (Table S20 in Additional file 6). Granulin in *O. viverrini *is defined as a proliferative growth factor and has been shown to be mitogenic at very low concentrations [14]. The genomic results provide strong evidence that granulin is also encoded in *C. sinensis*, and further work will determine its significance in the process of carcinogenesis. Thioredoxin peroxidase is characterized as an antioxidant enzyme ubiquitously expressed in the tissues of the liver fluke and in epithelial cells within the host bile duct [58]. Results suggest that thioredoxin peroxidase may play a significant role in protecting the parasite against damage and inducing inflammation in hosts. Our experiments have revealed the potential contribution of phospholipase A2 to hepatic fibrosis caused by *C. sinensis *infection. As an ES product, phospholipase A2 could bind to the receptor on the membrane of LX-2 cells [50]. Fatty acid binding protein is thought to have functions in lipid transport in parasites [59], but whether fatty acid binding protein is involved in carcinogenesis requires further clarification.

It has been acknowledged that liver fluke-induced CCA is a multifactorial pathological process resulting from infection-induced inflammation and the release of carcinogenic substances by parasites [14]. Both proteomic and transcriptomic approaches to the study of secreted and tegumental proteins have enhanced our understanding of the molecular mechanisms by which liver flukes establish a chronic infection, evade the host immune system and ultimately contribute to the onset of cancer [60]. However, the intrinsic molecular mechanisms involved in these processes remain obscure. Long-term hepatobiliary damage may result from multiple factors, including mechanical irritation of the epithelial cells, DNA damage from endogenous and exogenous carcinogens, and immunopathological processes directed by ES products and tegumental proteins. Moreover, increased concentrations of *N*-nitroso compounds in humans infected with liver flukes may contribute to the risk of developing CCA through the alkylation or deamination of DNA [54]. The results from our genomic study will help to elucidate previous hypotheses and aid us to explore more potentially important molecules associated with liver fluke-induced CCA.

## Conclusions

This study provides the fundamental biological characterization of the carcinogenic human liver fluke *C. sinensis*, which has large socio-economic and public health effects in Asian countries [1,2]. Recently, the advent of next-generation sequencing technology provided us with an unprecedented opportunity to obtain whole-genome sequence information for this neglected parasite. We report here the draft genome of *C. sinensis *based on DNA isolated from a single individual parasite. Briefly, our work contributes needed knowledge to decode the mechanisms underlying energy metabolism, developmental biology and pathogenesis in *C. sinensis*. Large pathogenic molecules involved in liver fluke-induced hepatobiliary disease have been discovered [6]. Numerous multifunctional secreted proteases and tegumental proteins have been highlighted for further study as vaccine and drug targets. In conclusion, the results presented here characterize the genomic features of *C. sinensis *and reveal the evolutionary interplay between parasite and host. We believe that the discoveries made in the *C. sinensis *genome project will be quite valuable for the prevention and control of this liver fluke.

## Materials and methods

### DNA library construction and sequencing

Adult *C. sinensis *flukes were isolated from cat livers (Henan Province, China) and rinsed several times with phosphate-buffered saline. A single adult was chosen for genomic DNA extraction using phenol.

Two short-insert (350 bp and 500 bp) DNA libraries were constructed according to the Paired-End Sample Preparation Guide (Illumina, San Diego, CA, USA). Briefly, we nebulized 2.5 μg of DNA with compressed nitrogen gas, then polished the DNA ends and added an 'A' base to the ends of the DNA fragments. Next, the DNA adaptors (Illumina) were ligated to the above products, and the ligated products were purified on a 2% agarose gel. We excised and purified gel slices for each insert size (Qiagen Gel Extraction Kit; QIAGEN Co., Ltd, Shanghai, China). Two DNA libraries were amplified using the adaptor primers (Illumina) for 12 cycles, and fragments of approximately 450 bp and 600 bp (inserted DNA plus adaptors) isolated from agarose gels.

We performed cluster generation on the cBot (Illumina), following the cBot User Guide. Then, we performed a paired-end sequencing run on the Genome Analyzer *IIx *(Illumina) according to the user guide. A total of 188.6 million raw reads (115 bp each) were obtained. FastQScreen [61] was used to screen out Illumina adaptors and other contaminating sequences. After masking adaptor sequences and removing contaminated reads, clean reads were processed for computational analysis.

### RNA library construction and sequencing

Adult *C. sinensis *flukes were isolated from cat livers (Guangdong Province, China) and rinsed several times with phosphate-buffered saline. Twenty flukes were pooled and total RNAs were extracted using the standard TRIZOL RNA isolation protocol (Invitrogen, Carlsbad, CA, USA).

For high-throughput sequencing, the sequencing library was constructed by following the manufacturer's instructions (Illumina). Fragments of 300 bp were excised and enriched by PCR for 18 cycles. Then, we performed a paired-end sequencing run on the Genome Analyzer *IIx *(Illumina) according to the user guide. After masking adaptor sequences and removing contaminated reads, a total of 31,965,154 clean paired-end reads (2 × 75 bp) were processed for scaffolding.

### Sequence assembly and mapping

We used the *k*-mer method [62,63] to estimate genome size. We obtained the 17-mer depth distribution with Soapdenovo [64] and ABySS [65]. The real sequencing depth (C) was correlated with the peak of the 17-mer frequency (C_*k*-mer_), read length (L) and *k*-mer length (K) in the formula:

$${\text{C}}_{k-\text{mer}}=\text{C}\times \left(\text{L}-\text{K}+1\right)∕\text{L}$$

Then, the genome size was estimated from total sequencing length and sequencing depth.

Clean reads were trimmed to 103 bp to minimize problems associated with low quality ends. We used the Celera Assembler [15] to assemble contigs and scaffolds, and we constructed super-scaffolds with the RNA-seq data using RNAPATH [17] (ERANGE module [66]).

We used Bowtie [19] to align trimmed reads to the assembled genome with no more than three mismatches and generated a sequence alignment/map (SAM) file. Reads that matched repetitive sequences were filtered out. We converted the SAM file to a GLF file using SAMtools [67] and called variants with glfSingle [68] with the following parameters: (i) the coverage depth of a single base must be 10× to 60×; (ii) the root mean squared (RMS) mapping quality score of overlapping reads must be at least 99; and (iii) the posterior probability threshold is 0.999.

### Repeat annotation

Known repetitive elements were identified using RepeatMasker [69,70] with the Repbase database [71,72] (version: 2009-06). A *de novo *repeat library was also constructed by using RepeatModeler, which contains two *de novo *repeat finding programs (RECON [23] and RepeatScout [24]). We used default parameters and generated consensus sequences and classification information for each repeat family. Then, we ran RepeatMasker on the genome again using the repeat library built with RepeatModeler.

### Gene model annotation

#### GeneWise

Predicted proteins from *S. japonicum *and *S. mansoni *were aligned to *C. sinensis *to identify conserved genes. Because GeneWise [73] is time consuming, schistosome proteins were first aligned with the *C. sinensis *genome using genBlastA [74]. Subsequently, we extracted matched genomic regions and used GeneWise to identify exon/intron boundaries.

#### Augustus and Genscan

Augustus [75] was run with the gene model parameters tuned for *Schistosoma*. Genscan [76] was run using the model parameters for human.

#### *C. sinensis *ESTs

cDNA libraries from *C. sinensis *metacercaria and adults were constructed using the standard Trizol RNA isolation protocol (Invitrogen), and the two libraries yielded 9,455 and 2,696 EST sequences, respectively. We also downloaded 2,970 existing EST sequences from the NCBI dbEST database. In addition, 574,448 EST sequences [3] were produced using the Roche 454 platform. We mapped all of the EST sequences to the *C. sinensis *genome with GMAP [77].

#### Integration of resources using EvidenceModeler

Gene predictions generated by Augustus and Genscan, spliced alignments of *S. japonicum *and *S. mansoni *proteins and EST alignments from *C. sinensis *were integrated with EvidenceModeler [78].

#### Protein domain analysis

InterProScan [79] was run on all *C. sinensis*, *S. japonicum *and *S. mansoni *predicted protein sequences. Matches tagged as 'true positive' (status 'T') by InterProScan were retained. InterPro domain information for five other species (*C. elegans*, *D. melanogaster*, *D. rerio*, *G. gallus *and *H. sapiens*) was downloaded from Ensembl BioMart (Ensembl version 60) [80].

#### Functional annotation

We mapped the *C. sinensis *reference genes to KEGG [81] pathways by BLAST (e-value < 1e-5). BLAST searches against the Swiss-Prot database and NCBI non-redundant database (e-value < 1e-5) were conducted to provide comprehensive functional annotation.

#### CEGMA validation

The CEGMA [27] set of 458 core eukaryotic genes was used to evaluate the completeness of the predicted gene models using the GenBlastA [74] program with default parameters.

### Gene family construction

Genes were clustered according to sequence similarity. We selected nine species in which to analyze gene families. The eight genomes were *C. sinensis*, *S. japonicum*, *S. mansoni*, *C. elegans*, *D. melanogaster*, *A. gambiae*, *D. rerio*, *G. gallus *and *H. sapiens*. Additional file 7 shows the sources of sequence data used in the present study [80,82-84]. For each gene, the longest protein product was used for alignment purposes. The peptide sequences were first aligned to other sequences from the same genome using BLAST. Hits with e-value < 1e-10 were used for clustering by Markov clustering [85] (the parameter -I was set to 6).

### Non-coding RNA annotation

The rRNA fragments were identified by aligning *C. sinensis *rRNA sequences from the NCBI Nucleotide database to the draft genome. The tRNA genes were found by running tRNAscan-SE [86] with eukaryote parameters. Other non-coding RNAs, including miRNAs, small nuclear RNAs and H/ACA-box small nucleolar RNAs, were identified by searching the Rfam database [87] with the software tool Infernal 1.0 [88].

### Synteny with *S. japonicum *and *S. mansoni*

Seventy-nine scaffolds with length greater than 200 kb were selected to perform pairwise genome alignment with *S. japonicum *and *S. mansoni *using BLASTZ [89] with the following parameters: C = 2, T = 0, W = 6, H = 2000, Y = 3400, L = 6000 and K = 2200. The Chain/Net package was used for post-processing, including lavToPsl, chainMergeSort, chainPreNet, chainNet, netToAxt and axtToMaf, and so on. All three of the genomes were masked with RepeatMasker using the '-s' setting.

### Phylogeny reconstruction

We selected nine species to construct a phylogenetic tree: *C. sinensis*, *H. sapiens*, *G. gallus*, *D. rerio*, *D. melanogaster*, *A. gambiae*, *C. elegans*, *S. mansoni *and *S. japonicum*. For each species, the longest transcript model was chosen to represent each gene, and genes shorter than 30 amino acids were excluded [64]. BlastP was used to compare all orthologues of the *C. sinensis *protein sequences against a protein database built from the other eight species (e-value < 1E-10), and the Solar program was used to concatenate fragmentary alignments for each pair of genes [64]. Genes that aligned with more than one-third of another gene in the same species were considered multi-copy genes and excluded from the analysis.

In total, 93 genes with single-copy orthologues in all species were identified. Individual multiple amino acid sequence alignments for each gene were created with CLUSTALW [90]. Those alignments that lacked informative sites or had too many gaps were discarded. The remaining 44 genes were concatenated into a final alignment. Regions with many mismatches were also discarded to reduce alignment error. The best protein model was found by MEGA5 [91] and used in the following analysis. The phylogeny tree was constructed by maximum likelihood methods using both MEGA5 and PHYML [92], which independently reached the same topology (only the results obtained from MEGA5 are presented here). Bootstrap values were based on 1,000 replicates. Tajima's relative rate test [93] was performed for *C. sinensi*s and *S. mansoni *(or *S. japonicum*), with *D. rerio *(or any of the other five species) used as an out-group.

### Data accessibility

All of the genome shotgun and transcriptome data are available in the NCBI Sequence Read Archive [SRA: 029284 and 035384]. The assembled genome and gene models are available at [94]. The genome sequences can also be downloaded from the DNA Data Bank of Japan [DDBJ: BADR01000001-BADR01060778 (contigs) and DF126616-DF142827 (scaffolds)]. The genome is available at the NCBI [NCBI: 72781], and the sequences are also available, from GenBank [GenBank: BADR00000000.1].

## Abbreviations

bp: base pair; CCA: cholangiocarcinoma; DMRT1: mab-3 related transcription factor 1; ES: excretory-secretory; EST: expressed sequence tag; FASN: fatty acid synthase; KEGG: Kyoto Encyclopedia of Genes and Genomes; miRNA: microRNA; NCBI: National Center for Biotechnology Information; ORF: open reading frame; PKA: cyclic AMP-dependent protein kinase; SOX6: sex determining region Y-box 6.

## Competing interests

The authors declare that they have no competing interests.

## Authors' contributions

XBY designed the study. XYW and WJC prepared the DNA samples, interpreted the data and wrote the paper. YH prepared the DNA samples and generated figures and figure legends. JFS prepared the DNA samples. FL prepared the DNA samples, analyzed the genome data and interpreted the data. HLL and LG analyzed the genome data, interpreted the data and generated figures and figure legends. JTM, XLL, CHD, CHZ, XRL, XCH and JX wrote the paper. CL, YXF and LSH generated figures and figure legends. All authors read and approved the final manuscript.

## Supplementary Material

Additional file 1 **Genome assembly and genome features of *C. sinensis*. **Figure S1: 17-mer depth distribution of the sequencing reads. Figure S2: features of the assembled *C. sinensis *genome. Figure S3: distribution of heterozygosity in *C. sinensis*. Figure S4: protein domain analysis of *C. sinensis*, *S. mansoni*, and *S. japonicum*. Table S1: main features of *C. sinensis *genome sequencing data. Table S2: numbers of reads mapped to the assembled *C. sinensis *genome. Table S3: genome validation by PCR products. Table S4: genome validation by Sanger ESTs. Table S5: repeat composition of *C. sinensis *genome. Table S6: summary of predicted protein-coding genes by different methods. Table S7: statistics of the reliable gene set with homology, or functional annotation or putative full-length ORF support [95]. Table S8: numbers of homologous genes between the CEGMA set of 458 core eukaryotic genes and our gene models. Table S9: summary of gene families in several organisms. Table S10: summary of genes annotated by InterPro domains in several species. Table S11: summary of predicted non-coding RNA genes in the *C. sinensis *genome. Click here for file

Additional file 2 **Summary of *C. sinensis *miRNA precursors**. Click here for file

Additional file 3 **Detailed information on putative syntenic blocks of *C. sinensis *versus *S. japonicum *and *C. sinensis *versus *S. mansoni*, respectively**. Click here for file

Additional file 4 **Important metabolism pathways of *C. sinensis*. **Figure S5: the glycolytic pathway of *C. sinensis*. All the key enzymes required for glycolysis were identified, indicating that the glycolytic pathway of *C. sinensis *is intact. EC numbers marked in red indicate the presence of the genes in the genome of *C. sinensis*. Figure S6: the Krebs cycle of *C. sinensis*. The Krebs cycle of *C. sinensis *is intact, reflected by related key enzymes present in *C. sinensis *genome, demonstrating that the liver fluke can generate energy from aerobic or anaerobic metabolism. EC numbers marked in red indicate the presence of the genes in the genome of *C. sinensis*. Figure S7: the fatty acid metabolism pathway of *C. sinensis*. *C. sinensis *can metabolize fatty acids as all required enzymes in the fatty acid metabolism pathway have been discovered. EC numbers marked in red indicate the presence of the genes in the genome of *C. sinensis*. Figure S8: the fatty acid biosynthesis pathway of *C. sinensis*. Only three enzymes in the fatty acid biosynthesis pathway were identified, indicating that *C. sinensis *cannot synthesize endogenous fatty acids. EC numbers marked in red indicate the presence of the genes in the genome of *C. sinensis*. Click here for file

Additional file 5 **Comprehensive analysis of genes involved in fatty acid biosynthesis in *C. sinensis*, *S. japonicum *and *S. mansoni***. Click here for file

Additional file 6 **Key molecules of *C. sinenisis***. Table S12: glycolysis molecules of *C. sinensis*. Table S13: protease molecules of *C. sinensis*. Table S14: kinase molecules of *C. sinensis*. Table S15: phosphatase molecules of *C. sinensis*. Table S16: tegument molecules of *C. sinensis*. Table S17: ES molecules of *C. sinensis*. Table S18: host binding molecules of *C. sinensis*. Table S19: sex determination molecules of *C. sinensis*. Table S20: CCA-related molecules of *C. sinensis*. Click here for file

Additional file 7 **Sources of gene sets used for comparative analysis**. Click here for file
