# Supplementary material for: The draft genome of the carcinogenic human liver fluke Clonorchis sinensis
Source: Genome Biol. 2011 Oct 24;12(10):R107. doi: 10.1186/gb-2011-12-10-r107 (PMC3333777; doi:10.1186/gb-2011-12-10-r107)
Supplement: Additional file 1 — Genome assembly and genome features of C. sinensis. Figure S1: 17-mer depth distribution of the sequencing reads. Figure S2: features of the assembled C. sinensis genome. Figure S3: distribution of heterozygosity in C. sinensis. Figure S4: protein domain analysis of C. sinensis, S. mansoni, and S. japonicum. Table S1: main features of C. sinensis genome sequencing data. Table S2: numbers of reads mapped to the assembled C. sinensis genome. Table S3: genome validation by PCR products. Table S4: genome validation by Sanger ESTs. Table S5: repeat composition of C. sinensis genome. Table S6: summary of predicted protein-coding genes by different methods. Table S7: statistics of the reliable gene set with homology, or functional annotation or putative full-length ORF support [95]. Table S8: numbers of homologous genes between the CEGMA set of 458 core eukaryotic genes and our gene models. Table S9: summary of gene families in several organisms. Table S10: summary of genes annotated by InterPro domains in several species. Table S11: summary of predicted non-coding RNA genes in the C. sinensis genome. [file gb-2011-12-10-r107-S1.DOC]

**Genome assembly and genome features of *C. sinensis***


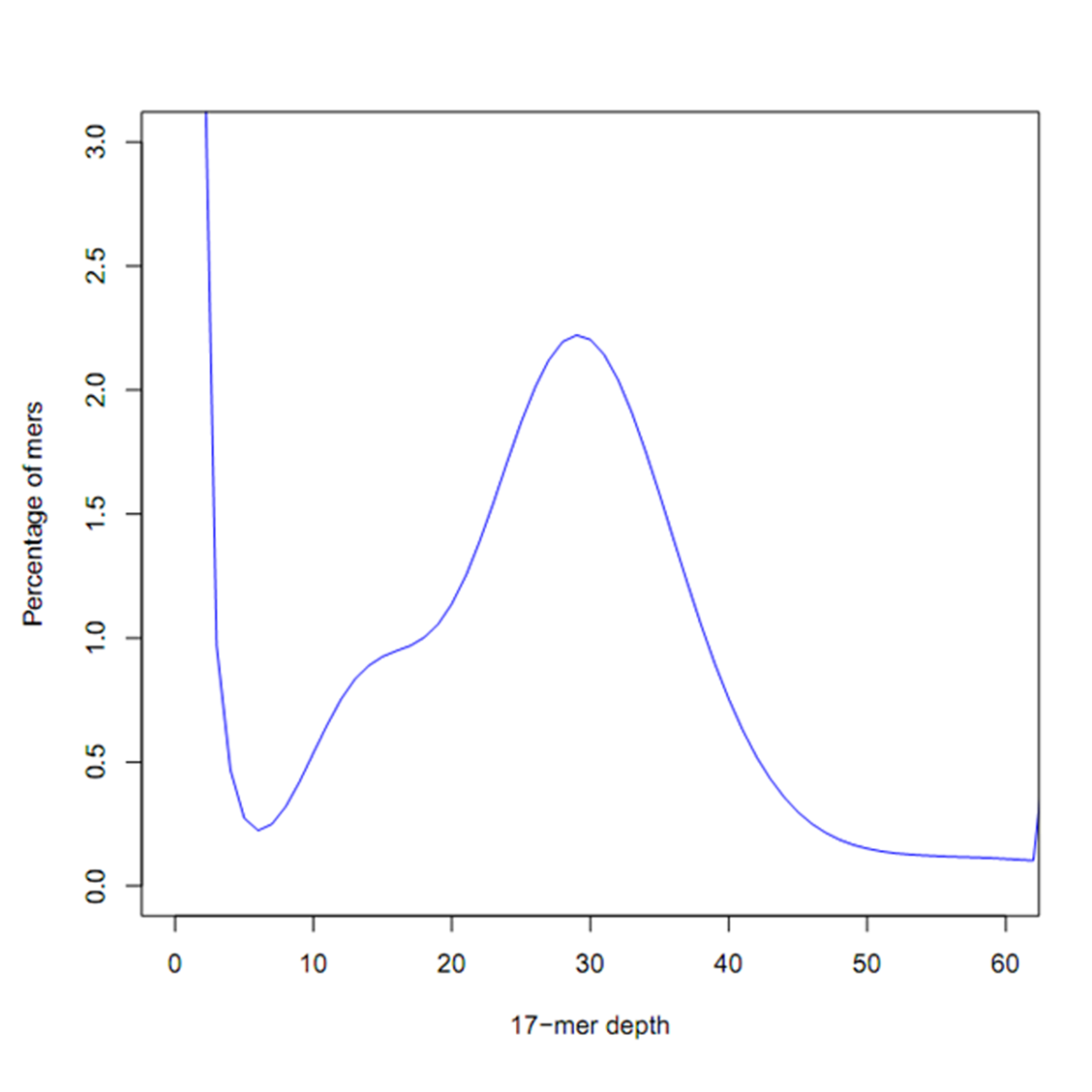


**Figure S1. 17-mer depth distribution of the sequencing reads. All sequencing reads were used to calculate the depth distribution with SOAPdenovo software. The peak was 29x. Genome size of *C. sinensis* was estimated as 644 M according to the distribution of 17-mers.**


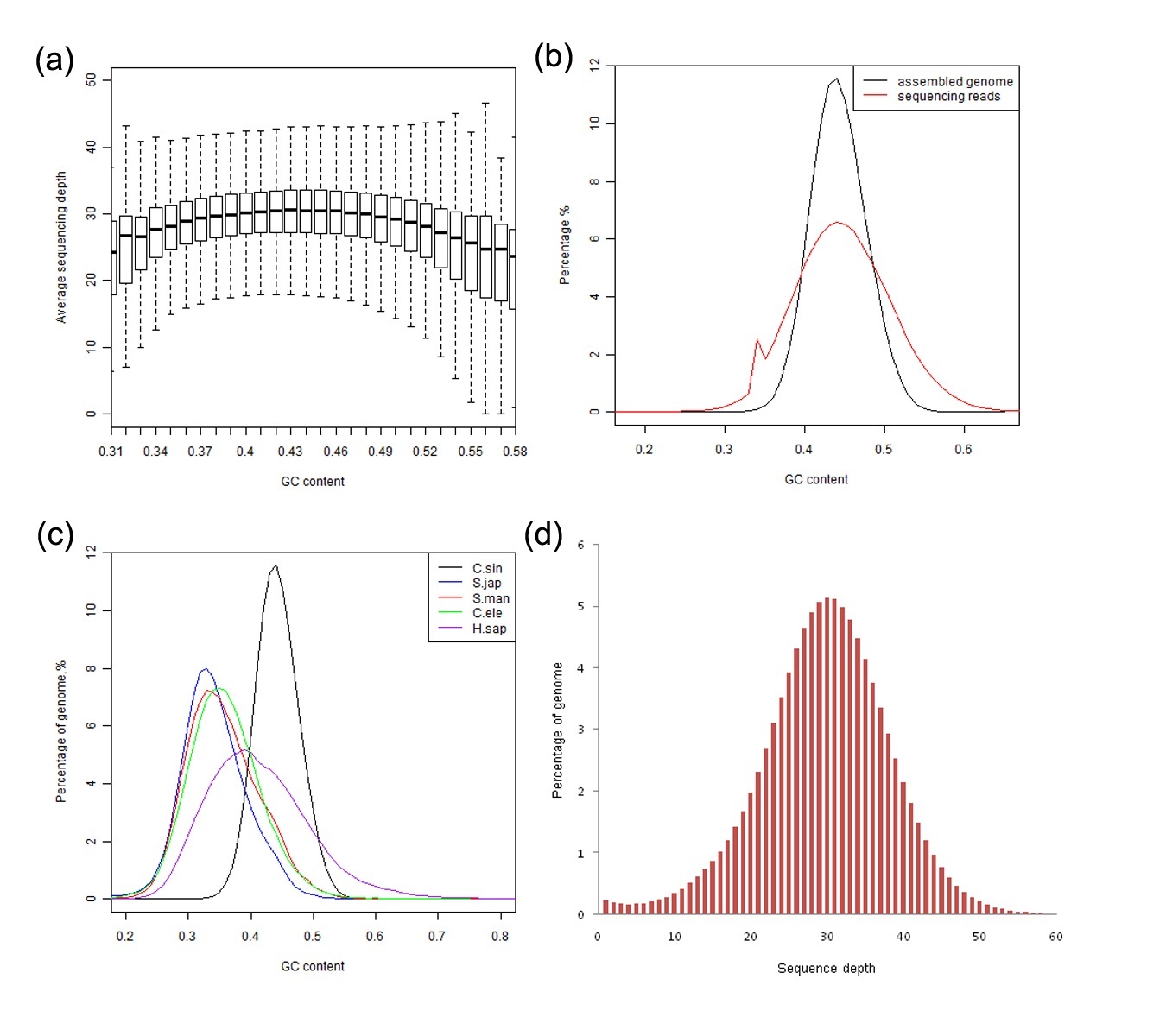


**Figure S2. The features of assembled *C. sinensis* genome. (a) Local GC content versus sequencing depth.** 500 bp non-overlapping sliding windows along with the assembled sequence were used to calculate GC content and average sequencing depth. (b) GC content of sequencing reads and assembled genome. The average GC content of sequencing reads (red) and assembled genome (black) was 44.38% and 43.85%, respectively. (c) **GC content distribution of *Clonorchis sinensis*, *Schistosoma japonicum*, *Schistosoma mansoni*, *Caenorhabditis elegans*, and *Homo sapiens.* (**d) **Distribution of sequencing depth of the assembled genome. The peak was 30x. 98% of genome was covered by more than 10 reads.**


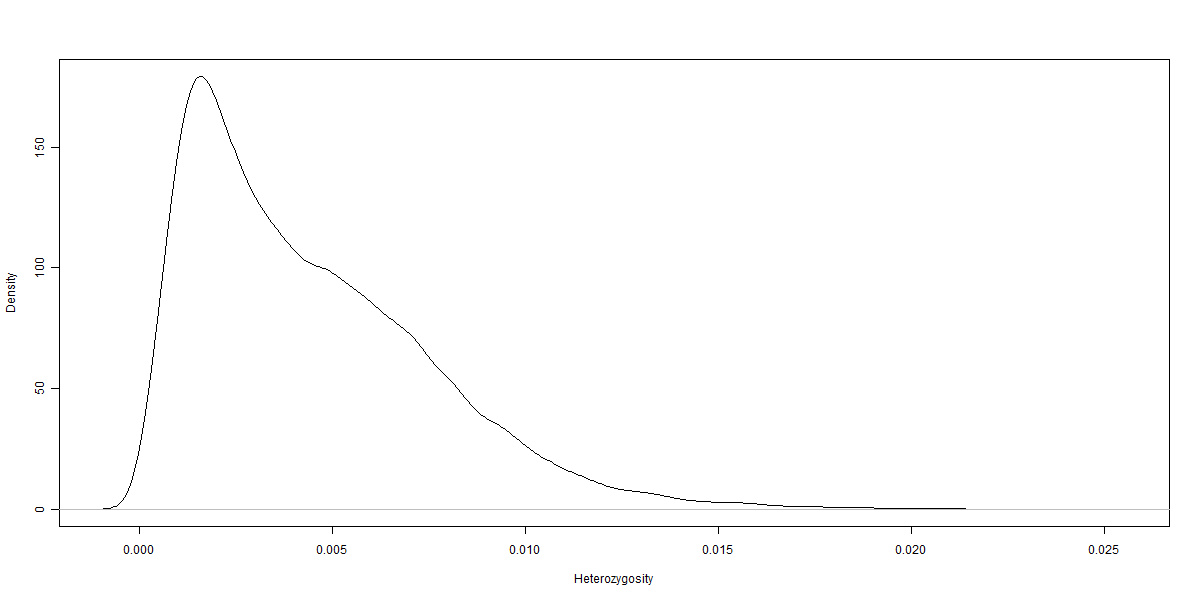


**Figure S3. Distribution of heterozygosity in *C. sinensis*. We used non-overlapping 10 kb windows and then calculated heterozygosity in each window.** The heterozygosity was approximately 0.4% for the whole genome**.**

**
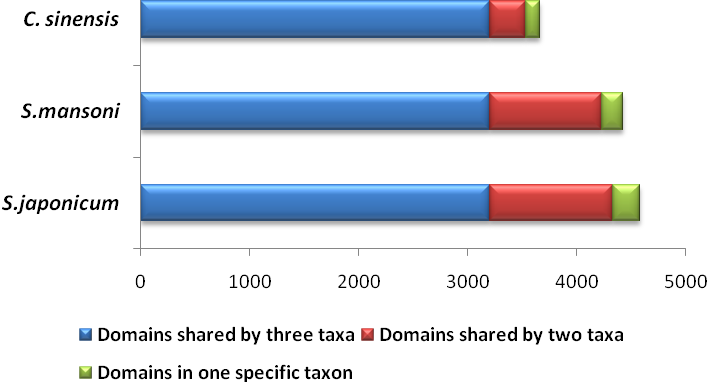
**

**Figure S4. Protein domain analysis of *C. sinensis, S. mansoni,* and *S. japonicum*. A total 5,027 protein domain types were detected in *C. sinensis*, *S. mansoni* and *S. japonicum*. 3,204 domain types were shared by all three flukes.**

**Table S1. Main features of *C. sinensis* genome sequencing data**

| **Insert size of libraries**  **(bp)** | **# of GA lanes** | **Avg. reads length (bp)** | **# of reads** | **Sum of reads length**  **(GB)** | **Sequence coverage**  **(X)^*^** | **Physical coverage**  **(X)^*^** |
| --- | --- | --- | --- | --- | --- | --- |
| **350** | **2** | **115** | **54,902,516** | **12.63** | **19.6** | **29.8** |
| **500** | **2** | **115** | **39,400,565** | **9.06** | **14.1** | **30.6** |
| **All** | **4** | **115** | **94,303,081** | **25.69** | **33.7** | **60.4** |

*We calculated sequence and physical coverage based on the estimated genome size of 644 M.

**Table S2. Numbers of reads mapped to the assembled *C. sinensis* genome**

|  | **# of mismatch** | **# of reads** | **% of reads** |
| --- | --- | --- | --- |
| **Unique-mapped** | **0** | **112,729,998** | **59.78** |
|  | **1** | **24,531,382** | **13.00** |
|  | **2** | **7,861,556** | **4.17** |
|  | **3** | **3,815,863** | **2.02** |
|  | **total** | **148,938,799** | **78.98** |
| **Multi-mapped** | **≤ 3** | **9,304,459** | **4.93** |
| **Un-mapped** |  | **30,344,904** | **16.09** |
| **Total** |  | **188,588,162** | **100** |

**Table S3. Genome validation by PCR products**

| **PCR products id** | **Primer** | **Length (bp)** | **Subject** | **Start** | **End** | **Identity** | **% of mismatch*** |
| --- | --- | --- | --- | --- | --- | --- | --- |
| **E02_340191.1.G1101244144.1.1-F(A3067)** | CAGCCAGAATACCCGAAGA | 575 | scf01432 | 56640 | 57214 | 572/575 | 0.52 |
| **E03_340191.2.G1101244145.1.1-R(A3068)** | TCGTAATTGGTTCCGCTCT | 568 | scf01432 | 57149 | 56582 | 565/568 | 0.53 |
| **E04_340191.3.G1101244146.2.2-F(A3069)** | CTGGGATGCTCCTTTATTCG | 668 | scf09369 | 7983 | 8650 | 667/668 | 0.15 |
| **E05_340191.4.G1101244147.2.2-R(A3070)** | TTCCACAATGCCTTTCAAG | 617 | scf09369 | 10712 | 10096 | 617/617 | 0.00 |
| **E06_340191.5.G1101244148.3.3-F(A3071)** | GGCAGTTGCTCGTAGACA | 372 | scf23554 | 7080 | 7451 | 367/372 | 1.34 |
| **E07_340191.6.G1101244149.3.3-R(A3072)** | TACTTGGCGGGAACTCTT | 327 | scf23554 | 7383 | 7057 | 324/327 | 0.92 |
| **E08_340191.7.G1101244150.4.4-F(A3073)** | ACGCTCCATCCATCGGAAAC | 296 | scf18672 | 18662 | 18957 | 295/296 | 0.34 |
| **E09_340191.8.G1101244151.4.4-R(A3074)** | TAGCCCAACCGGATCACTTA | 614 | scf18672 | 20224 | 19612 | 611/614 | 0.49 |
| **E12_340191.11.G1101244154.6.6-F(A3077)** | TTCCTCCGGTTGTGTACATTTCC | 325 | scf12717 | 32003 | 32327 | 325/325 | 0.00 |
| **F01_340191.12.G1101244155.6.6-R(A3078)** | CCAACTTAGCCGGAAACGAA | 398 | scf12717 | 34790 | 34393 | 398/398 | 0.00 |
| **F02_340191.13.G1101244156.7.7-F(A3079)** | TACAGTTCGGCGATTCCACC | 587 | scf10467 | 72792 | 73378 | 586/587 | 0.17 |
| **F03_340191.14.G1101244157.7.7-R(A3080)** | GACGGGTTGCTGGCACTA | 275 | scf10467 | 74567 | 74293 | 275/275 | 0.00 |
| **F04_340191.15.G1101244158.8.8-F(A3081)** | GACGGGCAGTAGATGAGG | 748 | scf00153 | 25286 | 26033 | 747/748 | 0.13 |
| **F05_340191.16.G1101244159.8.8-R(A3082)** | GTTGCTTACCGAACCAGA | 809 | scf00153 | 27238 | 26430 | 809/809 | 0.00 |
| **F06_340191.17.G1101244160.9.9-F(A3083)** | CCATCAGAGCTGCCTTGTC | 898 | scf24606 | 29208 | 30105 | 898/898 | 0.00 |
| **F07_340191.18.G1101244161.9.9-R(A3084)** | TCTTGGATGTCGGCGGTA | 899 | scf24606 | 30241 | 29343 | 899/899 | 0.00 |
| **H05_329431.1.G1101144271.14-3-3.14-3-3F(A2666)** | GTCGGATCCATGACTGAACGTGAGACTC | 807 | scf08144 | 11412 | 10607 | 805/807 | 0.25 |
| **H06_329431.2.G1101144272.14-3-3.14-3-3R(A2667)** | ATACTCGAGCTACGCCTTCTCCTCCG | 873 | scf08144 | 10572 | 11443 | 871/873 | 0.23 |
| **H07_329431.3.G1101144273.CYS.CYSF(A2668)** | ATAGGATCCATGACGTCAACACGTCTG | 494 | scf23554 | 7409 | 6916 | 490/494 | 0.81 |
| **H08_329431.4.G1101144274.CYS.CYSR(A2669)** | CGCCTCGAGTCAAAAATAATCCAACGG | 491 | scf23554 | 6996 | 7486 | 483/491 | 1.63 |
| **HB1-GAPDH.seq** | ACTGGGATCCGAGATGTCCAAACCTAAG | 870 | scf00518 | 299295 | 300014 | 868/870 | 0.23 |
|  | GCCCCTCGAGCCATTCTTCTTGAATTTA |  |  |  |  |  |  |
| **CSHN1-ACTIN.seq** | ATGGGTGATGAGGACGTTGCAGCT | 777 | scf24125 | 23701 | 24477 | 771/777 | 0.77 |
|  | CATGATCGAGTTGTACGTCGTCTC |  |  |  |  |  |  |
| **CSHN1-EF-1A.seq** | ACAGCACCACTCCTCCGTAT | 789 | scf23034 | 923 | 1711 | 787/789 | 0.25 |
|  | CCTCAGTCTTCTTGGCAACC |  |  |  |  |  |  |
| **CSHN1_tubulin.seq** | GCTTACTCATTCCCTTGGTG | 782 | scf02450 | 36135 | 35354 | 782/782 | 0.00 |
|  | ATCTCGTCCATACCTTCTCC |  |  |  |  |  |  |

***The proportion of mismatch sites between the genome and PCR products is 0.37% on average.**

**Table S4. Genome validation by Sanger ESTs**

|  | **Total #** | **Mapped # (%)** | |
| --- | --- | --- | --- |
|  |  | **All** | **Filtered*** |
| **ESTs from NCBI** | **2970** | **2741(92.3)** | **2659 (89.3)** |
| **Metacercaria ESTs^§^** | **9455** | **8230(87.4)** | **8109 (85.7)** |
| **Adult ESTs^§^** | **2696** | **2605(96.6)** | **2574 (95.5)** |
| **Total** | **15121** | **13576(89.8)** | **13342(88.2)** |

^§^ We sequenced metacercaria ESTs and adult ESTs and constructed metacercaria cDNA library and adult cDNA library in our laboratory.

***Sanger ESTs were anchored onto the genomic assemblies as spliced alignments using BLAT. In total, 13,342 out of 15,121 transcript sequences (88.2) could be mapped to the genomic sequence with a minimum alignment length of 100 nucleotides.**

**Table S5. Repeat composition of *C. sinensis*** genome

|  | **RepeatMasker** | | | **RepeatModeler** | | | **Total** | | |
| --- | --- | --- | --- | --- | --- | --- | --- | --- | --- |
|  | **Number of elements (#)** | **Length occupied (bp)** | **Percentage of sequence  (%)** | **Number of elements (#)** | **Length occupied (bp)** | **Percentage of sequence (%)** | **Number of elements (#)** | **Length occupied (bp)** | **Percentage of sequence (%)** |
| **SINEs** | **2074** | **190699** | **0.04** | **0** | **0** | **0** | **2074** | **190699** | **0.04** |
| **LINEs** | **1163** | **107552** | **0.02** | **151054** | **53385077** | **10.34** | **152217** | **53492629** | **10.36** |
| **LTR elements** | **52** | **2884** | **0** | **13850** | **5341271** | **1.03** | **13902** | **5344155** | **1.03** |
| **DNA elements** | **55** | **3606** | **0** | **5407** | **1859650** | **0.36** | **5462** | **1863256** | **0.36** |
| **Unclassified** | **1** | **52** | **0** | **373310** | **71661667** | **13.88** | **373311** | **71661719** | **13.88** |
| **Simple repeats** | **22360** | **1300419** | **0.25** | **1** | **24** | **0** | **22361** | **1300443** | **0.25** |
| **Low complexity** | **6227** | **206195** | **0.04** | **0** | **0** | **0** | **22361** | **206195** | **0.04** |
| **Total** | **31932** | **1811407** | **0.35** | **543622** | **132247689** | **25.61** | **575554** | **134059096** | **25.96** |

**Table S6. Summary of predicted protein-coding genes by different methods**

| **Gene set** |  | **# Number** | **Average length of gene length (bp)** | **Average length of CDS (bp)** | **# Exons per gene** | **Average length of exon (bp)** | **Average length of intron (bp)** |
| --- | --- | --- | --- | --- | --- | --- | --- |
| **Protein homology search** | ***S. jap*** | **6,562** | **1,748** | **462** | **1.57** | **293** | **1,456** |
|  | ***S. man*** | **8,603** | **2,319** | **492** | **1.7** | **288** | **1,767** |
| **Gene finder** | **Genscan** | **53,060** | **6,435** | **876** | **4.57** | **192** | **1,617** |
|  | **Augustus** | **10,085** | **7,293** | **999** | **3.58** | **279** | **2,299** |
| **EVM** | | **31,526** | **7,458** | **879** | **4.43** | **198** | **1,861** |

**Table S7. Statistics of reliable gene set with homology, or functional annotation or putative full-length ORFs support**

|  | **Database** | **# Number** | **Proportion (%)** |
| --- | --- | --- | --- |
| **Annotated** | **Swiss-Prot** | **8,888** | **54.6** |
|  | **KEGG** | **13,495** | **83.0** |
|  | **InterPro** | **6,847** | **42.8** |
|  | **GO** | **9,418** | **57.8** |
|  | **NR** | **13,648** | **83.9** |
|  | **Total** | **14,981** | **92.0** |
| **Un-annotated, but with *S. jap* or *S. man* homology** | | **744** | **4.6** |
| **Un-annotated, non-homology, but supported by putative full-length ORFs *** | | **533** | **3.3** |
| **Reliable gene set** | | **16,258** | **100** |

* Putative full-length ORFs were assembled by Newbler using 454 sequences and ORF were obtained by Orphelia [95]. The criterion of support by putative full-length ORFs is at least 50 amino acids alignment with > 90% identity.

**Table S8. Homolog gene numbers between CEGMA set of 458 core eukaryotic genes and our gene models**

| Alignment percent | >0% | >50% | >80% | >90% | >95% | Total |
| --- | --- | --- | --- | --- | --- | --- |
| Number of genes | 425 | 392 | 301 | 235 | 179 | 458 |
| Percent of genes | 92.8% | 85.6% | 65.7% | 51.3% | 39.1% | 100% |

**Table S9**. Summary of gene families in several organisms

| **Organism** | **Gene No.** | **MCL cluster No.*** | **Gene No./Clust No.** |
| --- | --- | --- | --- |
| ***C. sinensis*** | **16258** | **6910** | **2.35** |
| ***S. japonicum*** | **13469** | **8898** | **1.51** |
| ***S. mansoni*** | **11789** | **7313** | **1.61** |
| ***C. elegans*** | **20362** | **10180** | **2.00** |
| ***D. melanogaster*** | **13775** | **7640** | **1.80** |
| ***A. gambiae*** | **11971** | **6813** | **1.76** |
| ***D. rerio*** | **24004** | **7865** | **3.05** |
| ***G. gallus*** | **16704** | **8496** | **1.97** |
| ***H. sapiens*** | **21837** | **8841** | **2.47** |

* Gene families were estimated by gene clusters resulted from MCL algorithm according to sequence similarities of peptides (described in Methods Section).

**Table S10**. Summary of genes annotated by InterPro domains in several species

| **Species** | **Gene No.** | **Gene No. with protein domain** | **Domain types*** |
| --- | --- | --- | --- |
| ***C. sinensis*** | **16,258** | **6,847** | **3,675** |
| ***S. japonicum*** | **13,469** | **8,597** | **4,578** |
| ***S. mansoni*** | **11,789** | **6,898** | **4,422** |
| ***C. elegans*** | **20,224** | **13,470** | **4,129** |
| ***D. melanogaster*** | **13,804** | **10,737** | **5,012** |
| ***D. rerio*** | **24,020** | **21,207** | **5,787** |
| ***G. gallus*** | **16,736** | **12,854** | **5,361** |
| ***H. sapiens*** | **21,598** | **18,167** | **6,677** |

* Number of unique InterPro IDs annotating the genes of the species.

**Table S11. Summary of predicted non-coding RNA genes in the *C. sinensis* genome**

| **ncRNA Type** | **Copy #** | **Average Length**  **(bp)** | **Total length% (bp)** | **% of genome** |
| --- | --- | --- | --- | --- |
| **rRNA** | **7** | **338** | **2366** | **4.6e-4** |
| **tRNA** | **235** | **77** | **18,098** | **0.0035** |
| **snoRNA** | **509** | **77** | **39,008** | **0.0075** |
| **snRNA** | **169** | **80** | **13,603** | **0.0026** |
| **miRNA** | **858** | **64** | **55,333** | **0.01** |
