# Supplementary material for: The draft genome of the carcinogenic human liver fluke Clonorchis sinensis
Source: Genome Biol. 2011 Oct 24;12(10):R107. doi: 10.1186/gb-2011-12-10-r107 (PMC3333777; doi:10.1186/gb-2011-12-10-r107)
Supplement: Additional file 4 — Important metabolism pathways of C. sinensis. Figure S5: the glycolytic pathway of C. sinensis. All the key enzymes required for glycolysis were identified, indicating that the glycolytic pathway of C. sinensis is intact. EC numbers marked in red indicate the presence of the genes in the genome of C. sinensis. Figure S6: the Krebs cycle of C. sinensis. The Krebs cycle of C. sinensis is intact, reflected by related key enzymes present in C. sinensis genome, demonstrating that the liver fluke can generate energy from aerobic or anaerobic metabolism. EC numbers marked in red indicate the presence of the genes in the genome of C. sinensis. Figure S7: the fatty acid metabolism pathway of C. sinensis. C. sinensis can metabolize fatty acids as all required enzymes in the fatty acid metabolism pathway have been discovered. EC numbers marked in red indicate the presence of the genes in the genome of C. sinensis. Figure S8: the fatty acid biosynthesis pathway of C. sinensis. Only three enzymes in the fatty acid biosynthesis pathway were identified, indicating that C. sinensis cannot synthesize endogenous fatty acids. EC numbers marked in red indicate the presence of the genes in the genome of C. sinensis. [file gb-2011-12-10-r107-S4.DOC]

**Important metabolism pathways of *C. sinensis***


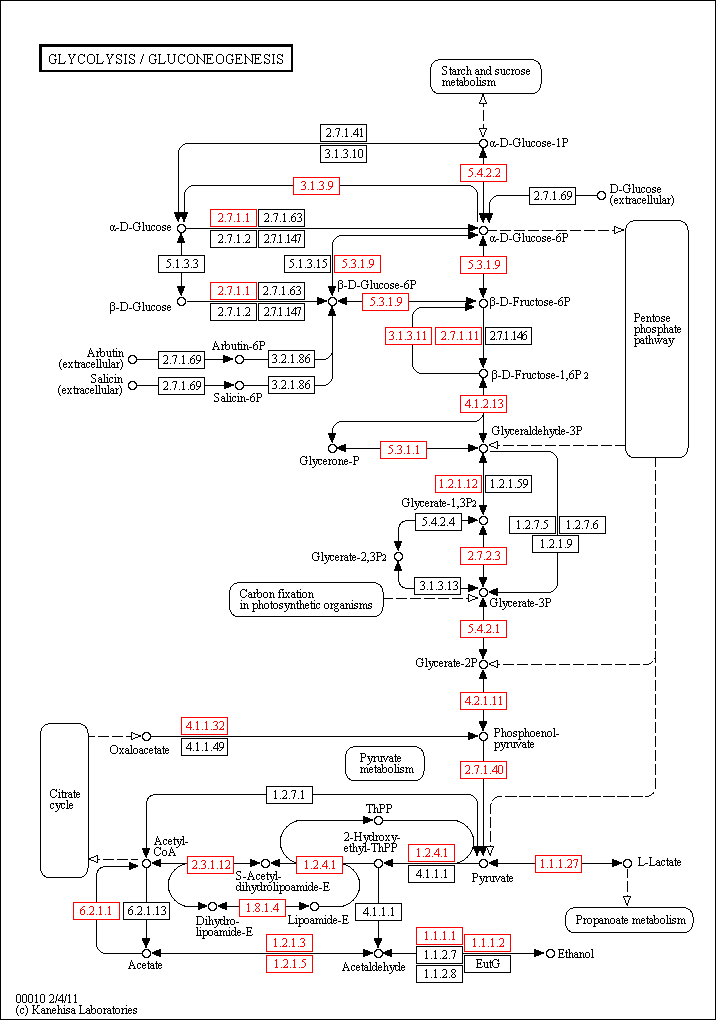


**Figure S5. The glycolytic pathway of *C. sinensis***. All the key enzymes required for glycolysis were identified, indicating that the glycolytic pathway of *C. sinensis* is intact. EC numbers marked in red indicated that the presence of genes in the genome of *C. sinensis*.


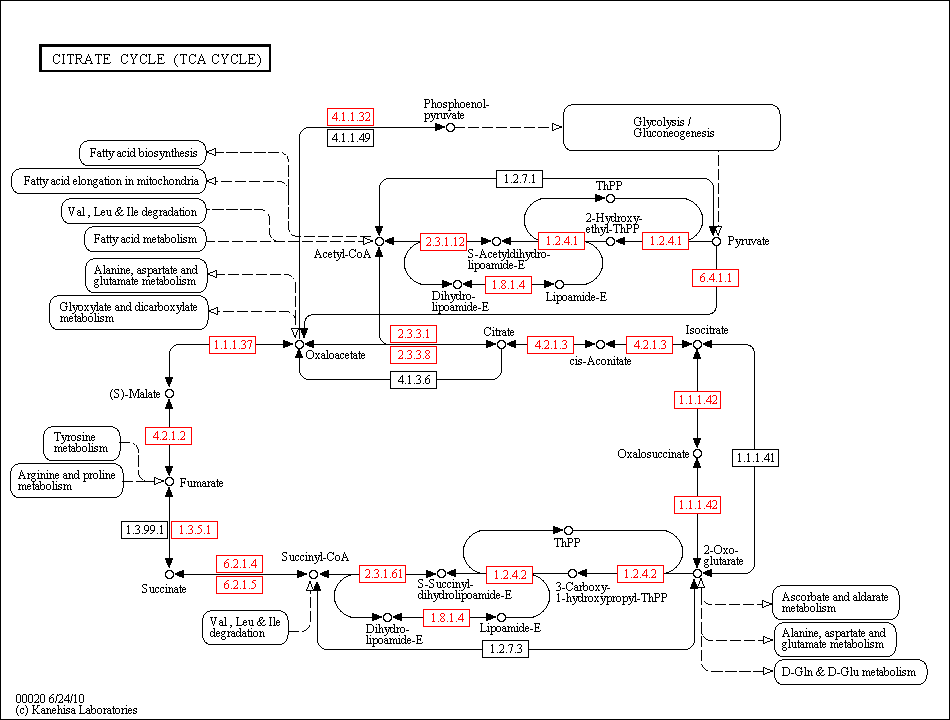


**Figure S6. The Krebs cycle of *C. sinensis.*** Krebs cycle of *C. sinensis* is intact, reflected by related key enzymes present in *C. sinensis* genome, demonstrating that energy for the liver fluke can be generated from aerobic metabolism or anaerobic metabolism. EC numbers marked in red indicated that the presence of genes in the genome of *C. sinensis*.


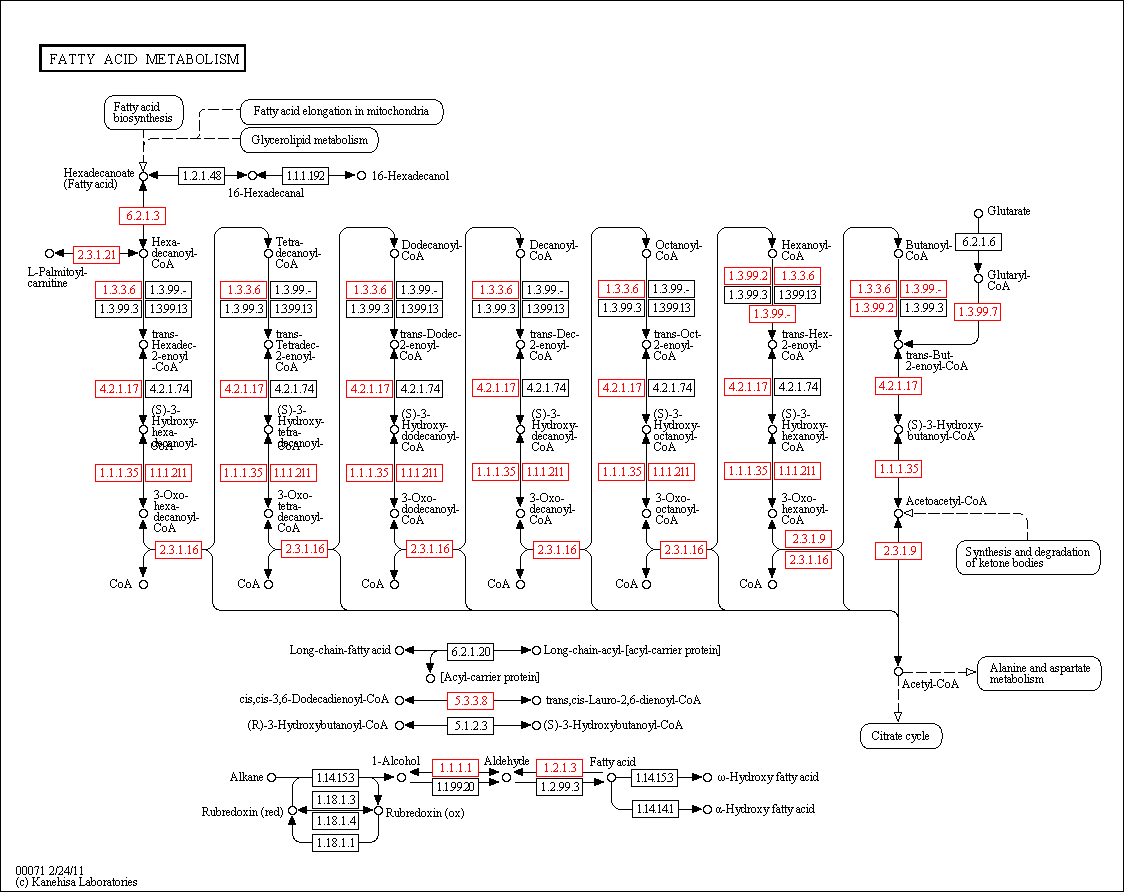


**Figure S7. The fatty acid metabolism pathway of *C. sinensis*.** *C. sinensis* is capable to make use of fatty acid for all required enzymes in fatty acid metabolism pathway have been discovered. EC numbers marked in red indicated that the presence of genes in the genome of *C. sinensis*.


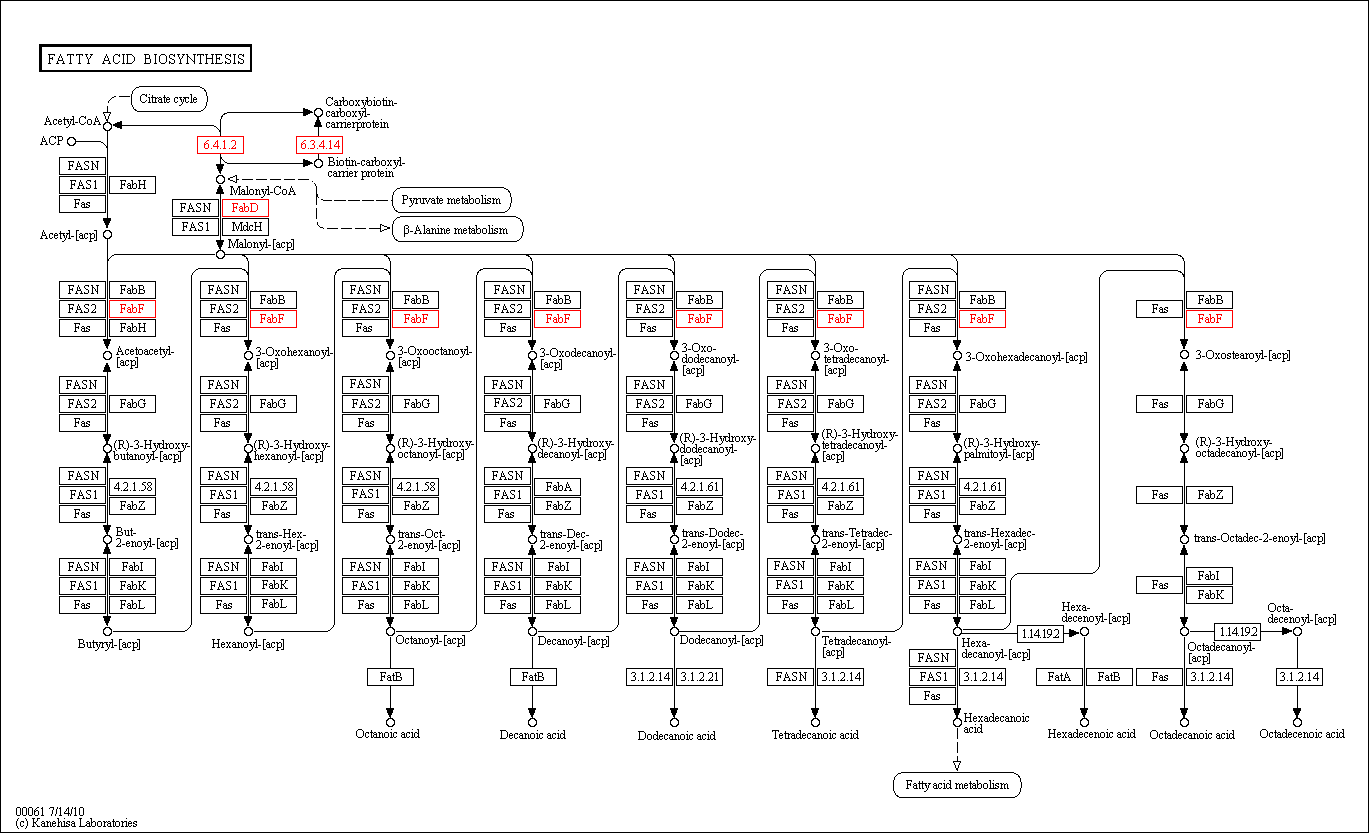


**Figure S8. The fatty acid biosynthesis pathway of *C. sinensis*.** Only three enzymes in the fatty acid biosynthesis pathway were identified, indicating that *C. sinensis* cannot synthesize endogenous fatty acids itself. EC numbers marked in red indicated that the presence of genes in the genome of *C. sinensis*.

acetyl-CoA carboxylase EC: 6.4.1.2, 6.3.4.14 KEGG: K11262 csin001769

FabD EC: 2.3.1.39 KEGG: K00645 csin013927

FabF EC: 2.3.1.179 KEGG: K09458 cisn013665
