# Supplementary material for: The draft genome of the carcinogenic human liver fluke Clonorchis sinensis
Source: Genome Biol. 2011 Oct 24;12(10):R107. doi: 10.1186/gb-2011-12-10-r107 (PMC3333777; doi:10.1186/gb-2011-12-10-r107)
Supplement: Additional file 7 — Sources of gene sets used for comparative analysis. [file gb-2011-12-10-r107-S7.DOC]

The resources of gene sets used for comparative analysis

| **Species** | **Source** |
| --- | --- |
| ***Homo sapiens*** | **[80]** |
| ***Gallus gallus*** | **[80]** |
| ***Danio rerio*** | **[80]** |
| ***Drosophila melanogaster*** | **[80]** |
| ***Anopheles gambiae*** | **[82]** |
| ***Caenorhabditis elegans*** | **[80]** |
| ***Clonorchis sinensis*** | **this study** |
| ***Schistosoma mansoni*** | **[83]** |
| ***Schistosoma japonicum*** | **[84]** |
